# Virtualization of Industrial Real-Time Networks for Containerized Controllers

**DOI:** 10.3390/s19204405

**Published:** 2019-10-11

**Authors:** Sang-Hun Lee, Jong-Seo Kim, Jong-Soo Seok, Hyun-Wook Jin

**Affiliations:** 1 Hyundai Mobis Co., Ltd., Yongin-si, Gyeonggi-do 16891, Korea; sanghun@mobis.co.kr; 2 LIG Nex1 Co., Ltd., Seongnam-si, Gyeonggi-do 13488, Korea; jongseo.kim@lignex1.com; 3 Electronics and Telecommunications Research Institute, Daejeon 34129, Korea; jsseok@etri.re.kr; 4 Department of Computer Science & Engineering, Konkuk University, Seoul 05029, Korea

**Keywords:** virtualization, controller area network, fieldbus, real-time, container

## Abstract

The virtualization technology has a great potential to improve the manageability and scalability of industrial control systems, as it can host and consolidate computing resources very efficiently. There accordingly have been efforts to utilize the virtualization technology for industrial control systems, but the research for virtualization of traditional industrial real-time networks, such as Controller Area Network (CAN), has been done in a very limited scope. Those traditional fieldbuses have distinguished characteristics from well-studied Ethernet-based networks; thus, it is necessary to study how to support their inherent functions transparently and how to guarantee Quality-of-Service (QoS) in virtualized environments. In this paper, we suggest a lightweight CAN virtualization technology for virtual controllers to tackle both functionality and QoS issues. We particularly target the virtual controllers that are containerized with an operating-system(OS)-based virtualization technology. In the functionality aspect, our virtualization technology provides virtual CAN interfaces and virtual CAN buses at the device driver level. In the QoS perspective, we provide a hierarchical real-time scheduler and a simulator, which enable the adjustment of phase offsets of virtual controllers and tasks. The experiment results show that our CAN virtualization has lower overheads than an existing approach up to 20%. Moreover, we show that the worst-case end-to-end delay could be reduced up to 78.7% by adjusting the phase offsets of virtual controllers and tasks.

## 1. Introduction

The contemporary industrial control systems comprise many sensors, actuators, and controllers connected through real-time networks. As the number of sensors and actuators in modern industrial plants increases drastically, the manageability of the controllers that directly interact with sensors and actuators in real-time becomes a serious concern. Accordingly, the demands for the flexibility with regard to hosting and consolidation of the controllers in large and complex industrial plants are constantly growing. For instance, to address the manageability and scalability in industrial control systems, there is an active movement to exploit cloud computing technologies in the infrastructure of smart manufacturing with the advent of the Industry 4.0 era [1,2,3].

In cloud computing, the virtualization is the key technology that provides the resource isolation and security between virtual machines [4]. Thus, it is expected that the industrial control software can also be efficiently deployed and executed in given computing resources by means of virtualization, while satisfying the requirements on security by preventing unauthorized resource access between virtual controllers. However, existing virtualization technologies in cloud computing do not support essential components of industrial control systems. It is particularly important to provide functional transparency and a real-time guarantee of industrial networks in virtualized environments. There were significant studies on network virtualization, but most of the existing research focused on the performance optimization of Transmission Control Protocol/Internet Protocol (TCP/IP) over Ethernet [5,6,7,8,9,10] or high-performance interconnects [11]. Although the Ethernet-based industrial networks, such as EtherCAT [12] and PROFINET [13], are emerging, traditional fieldbuses, such as Controller Area Network (CAN) [14], are still prevalent among the majority of control systems. CAN is a bus-based network standardized in ISO 11898. In order to support CAN in virtualized environments, we have to deal with following challenging issues:

- *Support for sharing of the network interface*: In order to allow several virtual controllers to share a physical CAN network interface in an isolated manner, the run-time support should be capable of multiplexing and demultiplexing the input/output (I/O) requests from multiple virtual controllers. However, the protocol stacks of CAN (i.e., CANopen [15]) implicitly assume that the CAN network interface can be dedicated to only one software controller.
- *Emulation of the media access control*: The characteristics of CAN are significantly different from general purpose networks. For example, the CAN message identifier is used in bus arbitration; that is, it is considered as a priority for bus arbitration. Thus, such characteristics have to be emulated in virtualized environments to preserve the behavior of controllers.
- *Low virtualization overheads*: As the traditional hypervisor-based virtualization (e.g., Xen [4], VMware [16], and VirtualBox [17]) adds significant run-time overheads, the operating-system (OS)-based virtualization (e.g., Container [18,19]) is emerging. Accordingly, we need a CAN virtualization technology that can be incorporated into the OS-based virtualization aiming to minimize the virtualization overheads.
- *Analysis of end-to-end delay*: In virtualized environments, multiple virtual controllers share the CPU resources; thus, the end-to-end delay of control loop highly depends on how the virtual controllers are scheduled. Therefore, we need a mechanism to analyze the worst-case end-to-end delay and minimize it to satisfy the requirements on real-time.

In this paper, we suggest a lightweight CAN virtualization technology for virtual controllers that are containerized with an OS-based virtualization. Our study mainly focuses on how to provide correct communication semantics and functionalities of industrial fieldbuses in OS-based virtualization, while providing low overheads and Quality-of-Service (QoS). The proposed scheme does not require any modifications of control applications and protocol stacks. There were also studies to virtualize CAN, but these were hardware-level approaches [20] or targeted the hypervisor-based virtualization [21]. We also suggest adjusting the phase offsets of virtual controllers and their tasks to minimize the end-to-end delay. By adjusting the execution point of tasks that perform communication over fieldbuses, we can improve the worst-case end-to-end delay. We implemented a simulation tool that finds a sub-optimal phase combination of virtual controllers and tasks. Although the phasing schemes were also discussed in other studies, these did not consider virtualized environments [22,23,24,25]. The performance measurement results show that our CAN virtualization technology hardly adds additional overheads and reports lower overheads than a hypervisor-based virtualization up to 20%. We also show that our phasing scheme can reduce the worst-case end-to-end delay by 47.0∼78.7%.

The rest of the paper is organized as follows: we discuss the related work in Section 2. We detail the suggested design of CAN virtualization and its implementation in Section 3. In this section, we present the device-driver-level CAN virtualization for containerized controllers and the simulation tool for optimal phasing of controllers and their tasks. The performance measurement results are presented in Section 4. Finally, we conclude this paper in Section 5.

## 2. Related Work

The virtualization technology provides multiple virtual platforms, each of which can run their own applications and system software on a single physical computing node. In legacy virtualization approaches, the software layer that provides the virtual machines is called hypervisor or Virtual Machine Monitor (VMM). A hypervisor can run on bare hardware (i.e., Type-1) or on top of an OS (i.e., Type-2). We call the OS running on the virtual machine as guest OS. In the Type-2 environment, the OS that hosts the hypervisor is called host OS. We can classify the virtualization technology into two: full-virtualization and para-virtualization. The full-virtualization allows the legacy software as either OS or applications to run in a virtual machine without any modifications. To do this, the hypervisors usually perform the binary translation and emulate every detail of physical hardware instruction sets. VMware [16] and VirtualBox [17] are examples of full-virtualization hypervisors. On the other hand, the para-virtualization requires modifications of guest OS in order to minimize the virtualization overhead. The hypervisors of para-virtualization provide guest OS with programming interfaces called hypercalls. Consequently, the para-virtualization presents better performance than full-virtualization. Xen [4] and XtratuM [26] are examples of para-virtualization hypervisors.

However, the para-virtualization also adds significant run-time overheads compared with the raw (i.e., non-virtualized) systems. The emerging OS-based virtualization [18] in which the OS instance is shared between the guest and the host domains does not induce significant overheads because the OS takes care of virtualization without extra software layers, such as hypervisor and multiple OS instances. In the OS-based virtualization, we call the guest domain as a container, and the OS has to guarantee the resource isolation between containers. The Linux kernel, for instance, provides *control group (cgroup)* and *name space* to guarantee the resource isolation with respect to resource usage and security, respectively [27].

The virtualization technology is mainly utilized in the cloud computing systems, but it also has very high potential of improving manageability and safety in industrial control systems. For example, the partitioning defined by ARINC-653 [28] and AUTOSAR [29] to provide temporal and spatial isolation between avionics and automobile applications can be ideally implemented by the virtualization technology [30]. In addition, there were efforts to host industrial control services in cloud infrastructures by virtualizing Programmable Logic Controllers (PLCs) and control networks [1,2,3]. These efforts correspond to the trend of running PLCs on open platforms such as PCs [31,32]. These studies have showed that the virtualization is a promising solution for providing infrastructure consolidation, manageability, resiliency, and security in industrial control systems. The existing studies, however, only targeted the Ethernet-based control networks, of which the virtualization technologies are already available; thus, there were no thorough discussions on how to host the traditional fieldbuses, such as CAN, in virtualized environments.

There has been significant research on network virtualization, which can be classified into network interface virtualization and Software-Defined Networking (SDN). Most of the existing network interface virtualization technologies focused on the performance optimization of TCP/IP over Ethernet interfaces [5,6,7,8,9,10] or high-performance interconnects such as InfiniBand [11]. A widely accepted approach is to provide multiple virtual network interfaces with the assistance of the network interface card. Since this approach requires the support from the network devices, it is not suitable to apply this to the fieldbus interface, which is not equipped with sufficient hardware resources to implement multiple virtual network interfaces. Though there was an architectural research on efficient network interface virtualization for CAN [20], it also highly depended on the assistance from a network interface card. To address the manageability and flexibility in the network architecture, the SDN technology that dissociates the control plane from data plane has been suggested [33]. There was a study to exploit SDN for control systems [34], but this also targeted only Ethernet because the existing SDN technology is limited primarily to IP-based networks.

Researchers also studied container scheduling for industrial IoT applications in cloud and fog computing environments [35,36]. However, they targeted soft real-time applications. There were studies to provide hard real-time scheduling in fog computing infrastructures [37,38], but these focused on task-level scheduling without consideration of container-level scheduling. In this paper, we study hierarchical CPU scheduling that performs task- and container-level scheduling for hard real-time control applications.

There is a lot of research on message scheduling [39,40,41,42,43] to meet the real-time constraints. Guaranteeing service-level real-time in a CAN-based networked control system is also studied [44]. There are several research works on synchronization between distributed controllers [45,46,47,48]. In this paper, we especially aim to adjust the phase offsets of virtual controllers and their tasks by means of the global clock and improve the worst-case end-to-end delay. Sung et al. [25] showed the possibility of synchronizing distributed control tasks by utilizing the global clocks of real-time control networks, but they considered only communication tasks while overlooking the preemption by higher-priority tasks. Kim and Kim [24], Kang et al. [23], and Lee at al. [22] exploited the global clock more positively to adjust task phases for isochronous control on EtherCAT. Craciunas et al. [49] tried to decide the optimal offset of tasks in terms of utility. However, previous studies did not consider the virtualized environments, where we have to deal with the phase of guest domains as well as the phase of tasks.

## 3. Virtualization of Controller Area Network

In this section, we suggest a lightweight virtualization of the CAN fieldbus for containerized virtual controllers. In addition, we implement a hierarchical real-time scheduler and a simulator to guarantee the real-time requirements of virtual controllers.

### 3.1. Design Issues

**Figure 1:**
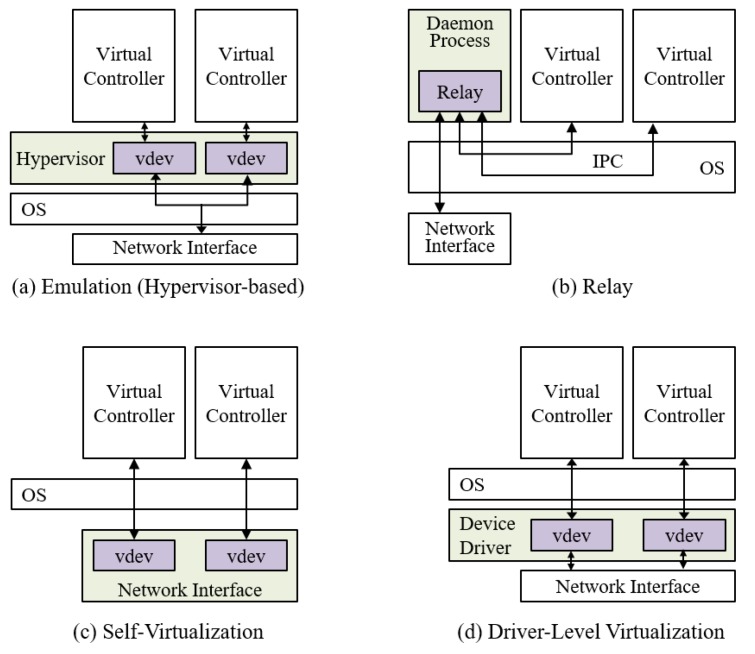
Alternatives for network interface virtualization.

We can consider four design alternatives for virtualization of industrial network interfaces as shown in Figure 1. In the emulation scheme (Figure 1a), the hypervisor emulates the target network interface and provides a channel to access the physical network interface that can be different from the target interface. However, as mentioned in Section 2, the hypervisor-based virtualization increases run-time overheads. Thus, we target the OS-based virtualization and consider virtual controllers as containerized instances. In the relay scheme (Figure 1b), a daemon process manages actual data transmission. The virtual controllers have to communicate with this daemon process through Inter-Process Communication (IPC) channels to send and receive messages. This solution is the only way to make the virtual controllers share the CAN interface without modifications of underlying system software or hardware. However, this approach not only requires modifications of control applications, but also induces a significant overhead due to IPC. The network interface capable of self-virtualization (Figure 1c) provides the virtual interfaces by itself. However, since each virtual interface is dedicated to a virtual controller, this alternative adds memory and computation overheads onto the network interface. The industrial network interfaces are usually equipped with a low-speed processing unit and low memory space; thus, many interfaces are not capable of accepting this design choice. The driver-level virtualization (Figure 1d) is somewhat similar with self-virtualization but virtual interfaces are provided by the device driver. Compared with relay and self-virtualization approaches, the driver-level virtualization is superior in both performance and resource requirements. We will describe the details of the driver-level virtualization in Section 3.2.

**Figure 2:**
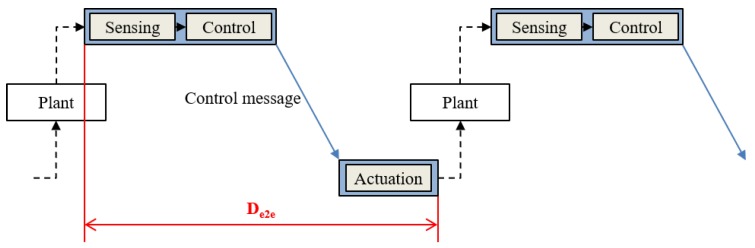
End-to-end delay of control loop.

**Figure 3:**
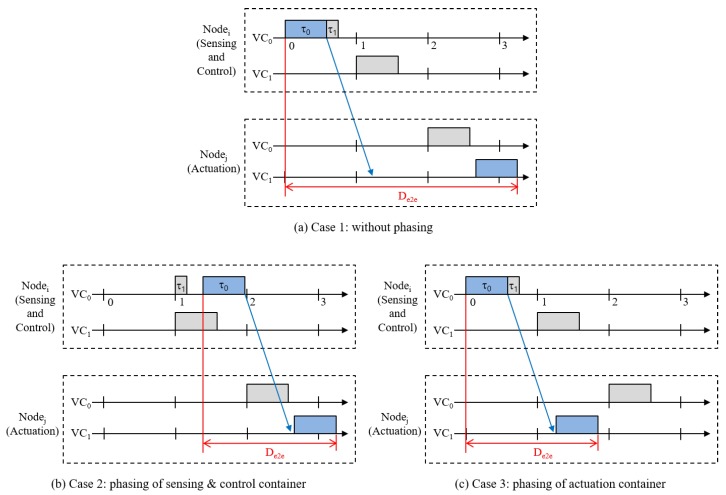
End-to-end delay and phase offsets.

To satisfy the real-time requirements of the virtual controllers, we have to pay careful attention to CPU scheduling. In virtualized environments, CPU scheduling is performed in a hierarchical manner [50]; first, the scheduler assigns the CPU resources to a container; then, the tasks that belong to the container are scheduled within the limit of the CPU resources assigned to the container. Legacy OS in cloud systems, however, focus on limiting the resource usage of containers rather than guaranteeing resources [51]. For instance, Linux keeps track of the resource usage of containers and throttles a container’s resource usage if that container exceeds the limit. That is, Linux does not guarantee resources but limits those. Thus, it is difficult to guarantee the deadlines of real-time applications. Our hierarchical scheduler provides the resource reservation based on a periodic execution model and guarantees the deadlines of hard real-time tasks. We will describe the implementation of the hierarchical real-time scheduler in Section 3.3. The end-to-end delay denoted as $D_{e2e}$ in Figure 2 is defined as the time from the beginning of the task that performs sensing and control to the completion of the task that performs actuation. For the sake of simplicity, we assume that the sensing and control operations are performed by a single task [24]. The sensing and control task sends control messages periodically to the actuation task. Since the tasks of the virtual controllers communicate through control networks, it is especially critical to decide when the tasks are scheduled and take part in communication. The simple examples in Figure 3 show how CPU scheduling impacts end-to-end delay. In these examples, $Node_{i}$ runs two virtual controllers denoted as $VC_{n}$. $VC_{0}$ comprises two tasks, $\tau_{0}$ and $\tau_{1}$. $\tau_{0}$ senses the plant, decides the control, and sends a control message to the actuation task running on $Node_{j}$. $\tau_{1}$ can be a Human–Machine Interface (HMI) task, for example. We assume that the sensing and control task, $\tau_{0}$, sends a message to the network at the finish time and the actuation task receives a message at the start time. In Case 1 (Figure 3a), the CPU resource of $Node_{i}$ is assigned to $VC_{0}$ in time slot 0 and two tasks are executed in succession. However, the actuation task on $Node_{j}$ runs long after the message arrives. Thus, the end-to-end delay becomes large. If $\tau_{0}$ decides a precise control based on the current situation of the plant, the control executed at $Node_{j}$ after a significant delay may not correspond to the situation at that time. In Case 2 (Figure 3b), if the period of $VC_{0}$ of $Node_{i}$ is two time slots, we can delay the execution of tasks in $VC_{0}$ of $Node_{i}$ to time slot 1 in which the release point of $\tau_{0}$ is delayed even more. This results in a less end-to-end delay than Case 1, while still guaranteeing the deadlines of containers and tasks. In this paper, we define the *phase offset* as an intentional delay of execution of containers and tasks. We can also adjust the phase offset of the actuation container as shown in Case 3 (Figure 3c), where the release point of the actuation task is advanced from time slot 2 to 1. To find a sub-optimal combination of phase offsets, we implement a simulator, which will be detailed in Section 3.4.

### 3.2. Driver-Level CAN Virtualization

**Figure 4:**
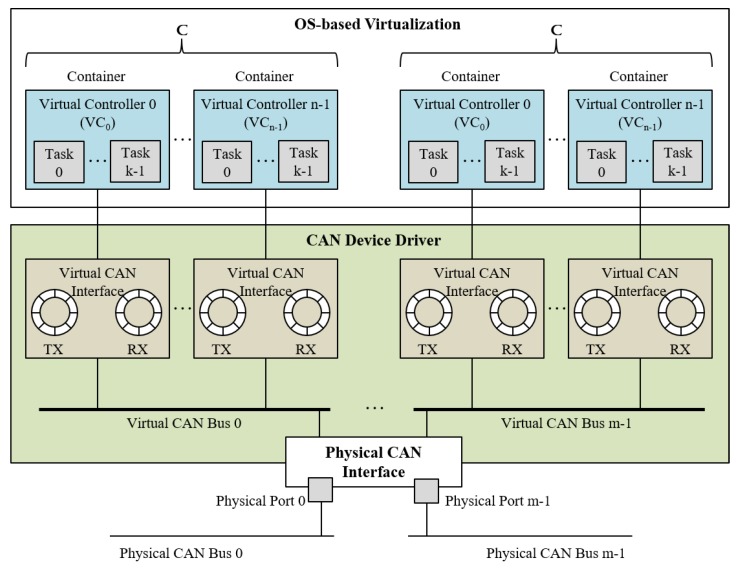
Driver-level Controller Area Network (CAN) virtualization.

As discussed in Section 3.1, the driver-level virtualization is more beneficial than the other design alternatives when considering transparency, performance, and resource requirements all together. Therefore, in this paper, we suggest the driver-level virtualization of CAN and study its implementation issues in detail. Figure 4 shows the suggested design. In the device driver, there are two main components to provide the CAN virtualization: *virtual CAN interface* that emulates the behavior of the CAN network interface and *virtual CAN bus* that emulates the media access control. A virtual controller owns exclusively an instance of the virtual CAN interface and is connected to a virtual CAN bus.

To provide functional transparency for virtual controllers, we have to emulate the inherent features and characteristics of CAN in the virtualized environments. The header of the CAN message includes the 11-bit message identifier, which specifies the class of information the CAN message represents (e.g., speed or torque of motors). The information that a specific identifier represents can vary from system to system and is determined at the system design phase. The controllers broadcast CAN messages tagging a message identifier according to its assignment rule and receive messages by specifying interesting message identifiers. It is to be noted that a CAN message specifies neither source nor destination and is simply broadcast to all nodes in the same bus. The CAN device drivers and CANopen allow only one task to receive messages of a specific identifier. If different tasks running on the same node wish to receive the CAN messages of the same identifier, it is not guaranteed that all tasks receive the messages properly. Only one task that issues the receiving operations before others can receive the messages. To overcome this limitation and host several virtual controllers, we provide the virtual CAN interfaces, which are created dynamically at the run time on demand and assigned exclusively to a virtual controller. A virtual CAN interface has a pair of send and receive queues and data structures for locking of the queues.

The CAN message identifier is also used in bus arbitration. When several controllers try to access the CAN bus simultaneously, the controller that tries to send the message with the lowest value of message identifier gains bus access. This means that the message identifier is considered as a priority for bus arbitration, where the lower identifier value, the higher priority. Since the virtual controllers are considered as separate CAN nodes in the virtualized environments, we need to arbitrate the bus access among the virtual controllers. We emulate the behavior of media access control in the virtualized environments by introducing the virtual CAN bus in the device driver as shown in Figure 4. When the physical CAN interface is able to send a message, the virtual CAN bus searches the send queues of all virtual CAN interfaces connected to the virtual bus, chooses the message that has the lowest identifier value, and sends it to the physical CAN bus. Moreover, the virtual CAN bus emulates the broadcast media. If a virtual controller sends a CAN message, it is sent out to the physical CAN bus but also delivered to the other virtual controllers connected to the same virtual bus. In non-virtualized environments, the propagation delay of messages on physical CAN bus is very small; thus, the geographical order of controllers on the bus may not be a critical issue. However, the virtual CAN bus copies messages to multiple virtual CAN interfaces to emulate the broadcasting media. The overhead of this copy operation can expand the time differences between message arrival points at local virtual CAN interfaces and remote CAN interfaces. Though we cannot provide a comparable latency to the physical bus, to mitigate the side effect of the copy overhead, the virtual CAN bus copies the message to the local virtual CAN interfaces and then sends the message to physical CAN bus. In a similar way, when a message is received from the physical CAN bus, the virtual CAN bus inserts the message to the receive queues of all virtual interfaces connected to the virtual bus. Since filtering of interesting messages is performed by the upper layers, we do not consider it at the virtual CAN bus.

The physical CAN interface in Figure 4 has multiple ports. The CAN interfaces in sensors or actuators usually have a single port, but we generalize our design and implementation for multiple ports because the computing nodes in cloud system can be equipped with a multi-port CAN interface. This allows a single computing node to host multiple sets of virtual controllers that use different CAN buses.

### 3.3. Hierarchical Real-Time Scheduling

As described in Section 3.1, we implement a hierarchical real-time scheduler for containerized controllers on Linux. The scheduler uses a fixed-priority scheduling algorithm (e.g., rate monotonic (RM) scheduling [52]) for both virtual controllers and tasks. We consider a set of periodic virtual controllers (i.e., $C=\{VC_{0},VC_{1},\cdots ,VC_{n-1}\}$) for each processor. Each virtual controller $VC_{i}$ is containerized with a different set of periodic tasks and uses a separate name space. $VC_{a}$ has a higher priority than $VC_{b}$ if a<b. We denote a virtual controller by $VC_{i}=\left(\Pi_{i},\Theta_{i},\Delta_{i},T_{i}\right)$, where $\Pi_{i}$ is the period, $\Theta_{i}$ is the time duration reserved for each period, $\Delta_{i}\;|\;0\leq \Delta_{i}<\Pi_{i}$ is the phase offset (i.e., temporal offset from a certain reference time), and $T_{i}$ is a set of periodic tasks, i.e., $T_{i}=\left\{\tau_{0}^{i},\tau_{1}^{i},\cdots ,\tau_{k-1}^{i}\right\}$. Task $\tau_{a}^{i}$ has a higher priority than task $\tau_{b}^{i}$ if a<b. A task is denoted as $\tau_{j}^{i}=\left(p_{j}^{i},e_{j}^{i},\delta_{j}^{i}\right)$, where $p_{j}^{i}$ is the period, $e_{j}^{i}$ is a range of execution time, and $\delta_{j}^{i}\;|\;0\leq \delta_{j}^{i}<p_{j}^{i}$ is the task-level phase offset. We assume that the relative deadline of each task is equal to its period. For the sake of simplicity, we also assume that a processor and a physical bus are dedicated to a set of VCs (i.e., C) launched by a tenant. If a processor is shared between multiple tenants that submit their Cs at arbitrary time points, it is difficult to analyze and guarantee the end-to-end delay on the fly. In addition, if a physical bus (and a virtual bus) is shared by different tenants, there can be conflicts between different definitions of message identifiers of disparate Cs, which results in malfunctions. Once a C finishes, it releases the processor and bus resources occupied so that another following C can be used. The applications at the plant level (e.g., Supervisory Control and Data Acquisition (SCADA) [53]) may consist of multiple Cs.

The hierarchical real-time scheduler is implemented as a daemon process. The scheduler suspends and resumes tasks by using signals. Once the system initialization is completed, the scheduler starts the timer. We use a global timer synchronized across distributed nodes. Although the fieldbuses, such as TTCAN [54] and EtherCAT [12], provide a global clock in distributed systems, we additionally implement a global clock in software for cases where a hardware global clock is not supported. Our software global clock is synchronized by using IEEE 1588 [55]. The scheduler releases a virtual controller $VC_{i}$ at $\Delta_{i}$. Then, the scheduler assigns the CPU resources to $VC_{i}$ for the duration $\Theta_{i}$ at every period $\Pi_{i}$. The task $\tau_{j}^{i}$ is released periodically after $\Delta_{i}+\delta_{j}^{i}$. The scheduler runs tasks based on their period $p_{j}^{i}$ and execution time $e_{j}^{i}$ within the CPU utilization of $VC_{i}$ (i.e., $\Theta_{i}/\Pi_{i}$).

**Figure 5:**
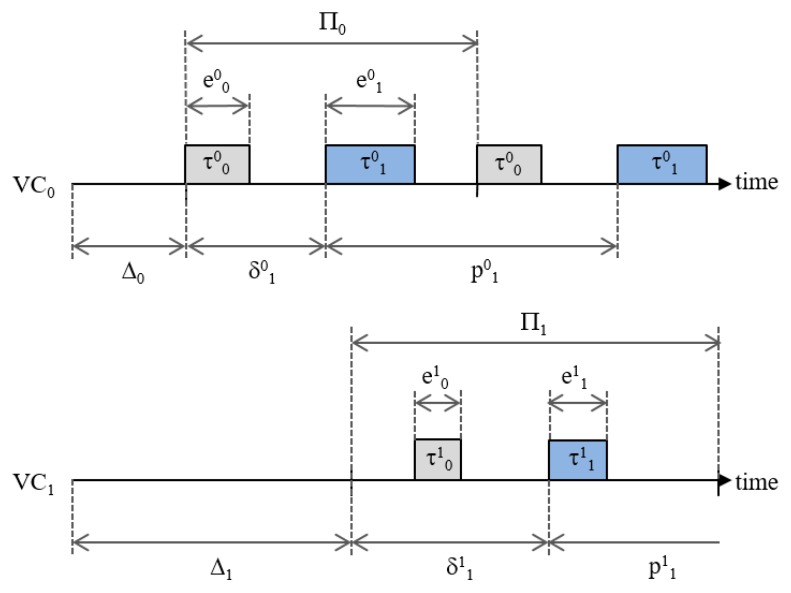
Hierarchical real-time scheduling.

Figure 5 shows how the phases $\Delta_{i}$ and $\delta_{j}^{i}$ decide the release point of a task. In this example, two virtual controllers (i.e., $VC_{0}$ and $VC_{1}$), each of which has two tasks, run on a single CPU. The first periods of $VC_{0}$ and $VC_{1}$ starts at $\Delta_{0}$ and $\Delta_{1}$, respectively. We assume that the highest-priority task of each virtual controller in this example has the zero phase offset (i.e., $\delta_{0}^{0}=0$ and $\delta_{0}^{1}=0$); thus, $\tau_{0}^{0}$ and $\tau_{0}^{1}$ are released as soon as the first period of each VC begins, whereas $\tau_{1}^{0}$ and $\tau_{1}^{1}$ start being released after $\Delta_{0}+\delta_{1}^{0}$ and $\Delta_{1}+\delta_{1}^{1}$, respectively. Since $VC_{0}$ has a higher priority than $VC_{1}$, the tasks of $VC_{1}$ are preempted by the tasks of $VC_{0}$. For example, we can see that the execution of $\tau_{0}^{1}$ is delayed because it is preempted by $\tau_{1}^{0}$. We will discuss the adjustment of phases in more detail in the next subsection.

### 3.4. Phasing of Virtual Controllers and Tasks

The worst-case end-to-end delay is an important metric of QoS for industrial control applications [56]. As we have discussed above, the worst-case end-to-end delay in virtualized environments is strongly influenced by the phase offsets of virtual controllers and tasks (i.e., $\Delta_{i}$ and $\delta_{j}^{i}$). Researchers tried to find an optimal combinations of phase offsets by suggesting either an online algorithm [23] or a simulation-based offline approach [22]. However, they did not consider the virtualized environments. In this paper, we implement a simulator that performs a discrete-event simulation and provides a sub-optimal phase combination for virtual controllers and tasks. It is to be noted that the phase combination suggested by the simulator does not hinder the deadline guarantee of containers and tasks.

**Figure 6:**
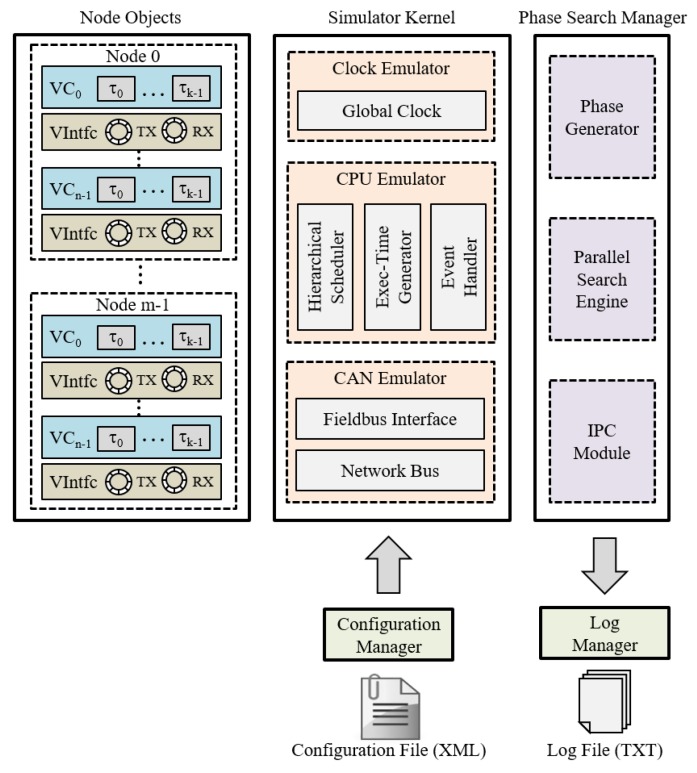
Simulator.

The simulator consists of configuration manager, node objects, simulator kernel, phase search manager, and log manager as shown in Figure 6. The configuration manager provides the user interfaces to configure simulation parameters, such as attributes of virtual controllers, tasks, and target fieldbus. The parameters are specified as an XML format and parsed by the configuration manager at the initialization phase. The node objects are created according to the simulation parameters. Each node object emulates a C. The simulation kernel emulates the run-time behavior of overall system by performing hierarchical CPU scheduling and message transmission. Since the execution time of tasks varies for every period in real systems, the exec-time generator emulates this by generating time values in the range of $e_{j}^{i}$ with a uniform distribution. In addition, the event handler and fieldbus interface components emulate system overheads, such as interrupt handling and Direct Memory Access (DMA). The simulation results are gathered by the IPC module and saved into text files by the log manager.

### 3.5. Implementation

We implemented the CAN virtualization at the Linux device driver of the PEAK-System CAN interface. In Linux, each port of the PEAK-System CAN interface is registered as a character device file. The device file is an abstraction implemented by OS to provide basic user-level operations on I/O devices. The applications and CANopen request the send and receive operations through the file I/O system calls, and the virtual file system of Linux internally calls the I/O functions provided by the device driver. In our implementation, a virtual CAN interface is created dynamically and destroyed at the run time when an application or CANopen calls the open() and close() system calls, respectively. A virtual CAN interface is exclusively assigned to a virtual controller. The send and receive operations are commenced by the ioctl() system call. In the case of send operation, the device driver first inserts the message to the send queue of the virtual interface, then seeks the message that has the highest priority (i.e., lowest message identifier number) across the send queues of the virtual interfaces, and not only sends the highest-priority message to the physical CAN interface, but also copies it into the receive queues of the other virtual interfaces. Regarding the receive operation, the interrupt handler in the device driver copies the received message into every receive queue of the virtual interfaces that are connected to the same virtual CAN bus. The ioctl() system call with receive command returns the message of desired identifier from the receive queue of the corresponding virtual CAN interface. We identify the corresponding virtual CAN interface by using the file descriptor passed by the system calls.

To force the container scheduler to behave like the RM scheduler, we set the period and runtime attributes of cgroup into $\Pi_{i}$ and $\Theta_{i}$, respectively. The Linux cgroup provides these attributes for a container that consists of the tasks scheduled by a Linux real-time scheduler (i.e., SCHED_FIFO or SCHED_RR). Then, it is guaranteed that the CPU usage of the container does not exceed the specified runtime for every period. We set the priority of tasks into the priority of the container to which the tasks belong. In addition, since the Linux real-time schedulers do not support periodic task scheduling, we implemented an overlay scheduler on Linux so that task scheduling can be performed with the RM algorithm. The overlay scheduler maintains a list of the container control blocks, each of which includes a list of task control blocks (i.e., $T_{i}$). A task control block includes the attributes of the task, such as period $p_{j}^{i}$ and worst-case execution time $Max(e_{j}^{i})$. The overlay scheduler selects a task to run from the current container based on the RM algorithm and suspends/resumes tasks by using signals, such as SIGSTOP and SIGCONT.

The current implementation of the simulation tool performs an exhaustive search to find a sub-optimal phase combination; that is, it investigates all possible combinations of phase offsets by shifting the phase offsets of containers and tasks by a given time unit. The simulation tool analyzes the worst-case end-to-end delay of a phase combination by simulating the control loops for a given number of iterations. Thus, the total simulation time and the accuracy of analysis highly depend on the time unit generating phase combinations and the number of iterations to simulate. Although the current implementation is enough to show the benefits of phasing for virtual controllers, further study is needed to reduce the number of phase combinations investigated and reduce the total simulation time without sacrificing the analysis accuracy. The simulator creates multiple processes as many as CPU cores to run simulations for different phase offsets in parallel. Once the phase offsets of containers and tasks are decided by the simulator, we set the parameters (i.e., $\Delta_{i}$ and $\delta_{j}^{i}$) of the hierarchical scheduler to the phase offset combination suggested.

### 3.6. Summary

In this subsection, we summarize how the suggested design and implementation address four challenges listed in Section 1. First, to support sharing of the CAN interface between virtual controllers, we proposed the virtual CAN interface and the virtual CAN bus as described in Section 3.2. A virtual controller is assigned a separate virtual CAN interface and allowed to use only the assigned virtual interface; that is, the other virtual interfaces of different virtual controllers are invisible as described in Section 3.5. The virtual CAN interfaces of virtual controllers in the same C are connected through a virtual CAN bus that takes care of multiplexing and demultiplexing of accessing to/from the physical CAN interface. Thus, we can isolate the communication of each virtual controller with respect to functionality, while allowing for sharing of the physical CAN interface.

Secondly, to emulate the media access control of CAN, the virtual CAN bus implements the bus arbitration and message broadcasting as described in Section 3.2. In CAN, the message identifier is used as the priority of messages in bus arbitration. As described in Section 3.5, the virtual CAN bus chooses the message that has the lowest identifier number from the send queues of the virtual CAN interfaces and sends it first. In addition, the virtual CAN bus implements message broadcasting by copying the message sent from a virtual controller or received from the physical CAN interface into the receive queues of the virtual interfaces connected to the same virtual CAN bus.

Thirdly, our driver-level CAN virtualization and hierarchical CPU scheduler support the OS-based virtualization, aiming for low virtualization overheads. As we have discussed earlier, the OS-based virtualization has lower overheads than the hypervisor-based virtualization. The CAN virtualization suggested in Section 3.2 is implemented at the CAN device driver; thus, it is transparent to the upper layers (i.e., OS kernel, CANopen, and applications) and harmonizes well with the OS-based virtualization. Moreover, the hierarchical CPU scheduler suggested in Section 3.3 is implemented for OS-based virtualization, targeting particularly containers and their tasks.

Finally, to analyze the end-to-end delay and enhance the worst-case end-to-end delay, we suggested a simulation tool and hierarchical real-time scheduling in Section 3.4 and Section 3.3, respectively. The simulation tool analyzes the end-to-end delay with different phase offsets of containers and tasks and suggests a phase offset combination that can provide a sub-optimal worst-case end-to-end delay. The hierarchical real-time scheduler implements a global timer to synchronize between distributed virtual controllers and can adjust phase offsets with the scheduling of periodic containers and tasks.

## 4. Experimental Results

In this section, we analyze the overheads of our driver-level CAN virtualization. In addition, we show how phasing of virtual controllers and tasks can improve the worst-case end-to-end delay in the virtualized environments.

### 4.1. Comparisons with Hypervisor-Based Virtualization

Our CAN virtualization targets the OS-based virtualization aiming for less overheads. To analyze the virtualization overheads, we measured the Round-Trip-Time (RTT) between two different physical nodes connected through a CAN bus. Each node was equipped with an Intel i5 processor and a PEAK-System CAN interface and installed the Linux operating system. We measured RTT between two nodes that sent and received the same size messages in a ping-pong manner repeatedly for a given number of iterations. In the experiments, we considered the message size only up to 8-byte because a CAN frame can convey 8-byte payload in maximum. We drew a comparison between three cases: (i) original setup without virtualization, (ii) OS-based virtualized environment, and (iii) hypervisor-based virtualized environment. The original setup shows the base performance to be compared with. The OS-based virtualization shows the performance of our design suggested in Section 3. To measure the CAN virtualization overheads in hypervisor-based virtualization, we used the implementation suggested by Kim et al. [21]. It is to be noted that we applied the hypervisor-based virtualization to only one node, while measuring RTT on the other node that is not virtualized because the hypervisor-based virtualization does not provide an accurate timer to guest domains. Similarly, we also applied the OS-based virtualization to only one node for fairness. In addition, in the experiments, the virtual controllers did not follow the periodic execution models described in Section 3.3 to measure pure communication overheads, removing the impact of phase offsets and variable execution time of tasks. We will analyze the impact of different phase offsets in the next subsection.

**Figure 7:**
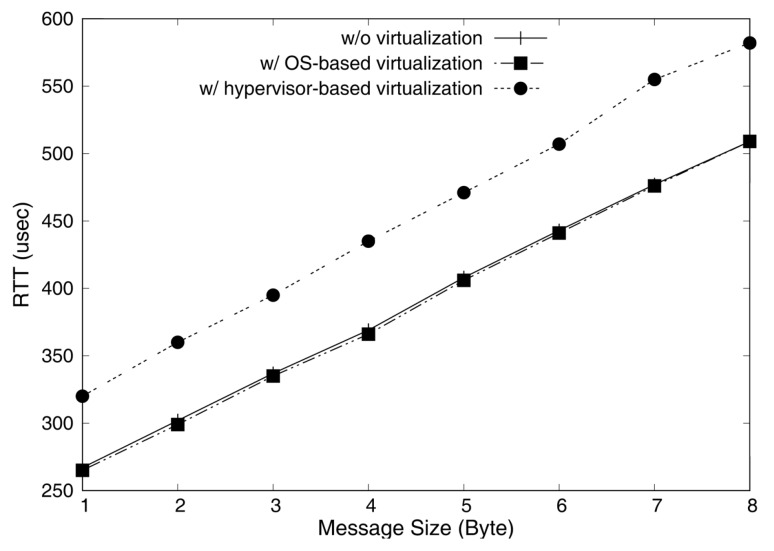
Average Round-Trip-Time (RTT) of different message sizes.

Figure 7 shows the average RTT for different message sizes. As we can see, the OS-based virtualization hardly adds additional overheads compared with the original setup without virtualization, whereas the hypervisor-based virtualization shows higher overheads up to 20%. The low overheads of the OS-based virtualization is due to not only the absence of a hypervisor, but also our lightweight driver-level CAN virtualization.

**Figure 8:**
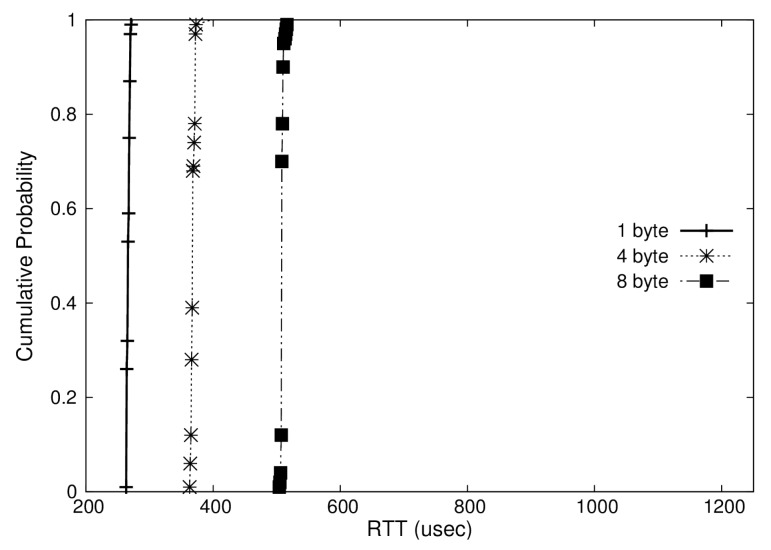
Cumulative Distribution Function (CDF) of RTT in a non-virtualized case.

**Figure 9:**
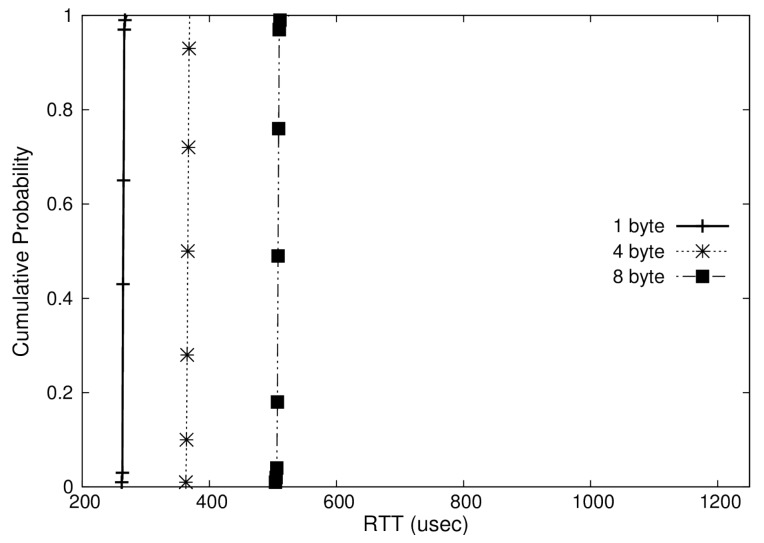
CDF of RTT in an operating-system(OS)-based virtualization case.

**Figure 10:**
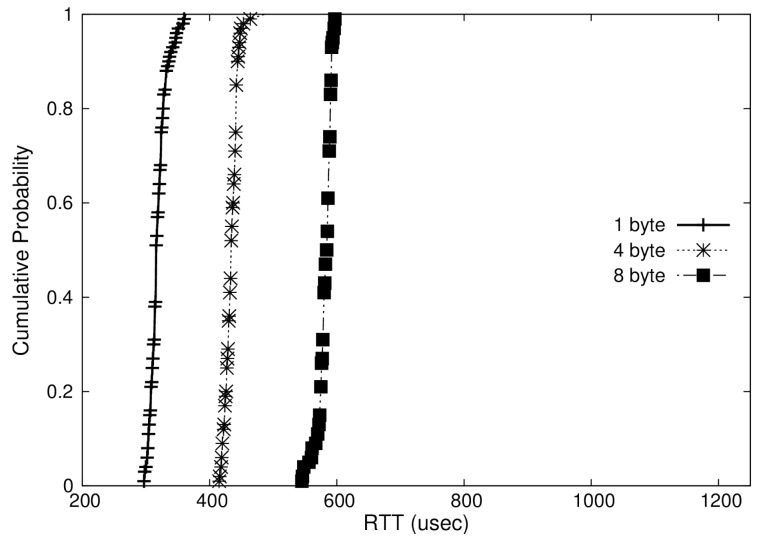
CDF of RTT in a hypervisor-based virtualization case.

In addition, we represented the distribution of RTTs measured in Cumulative Distribution Function (CDF) plots as shown in Figure 8, Figure 9 and Figure 10. These graphs show the RTTs of 1, 4, and 8-byte messages. As we have discussed, the OS-based virtualization shows a comparable performance to a non-virtualized environment, while the hypervisor-based virtualization shows higher overheads. Moreover, we can observe that the jitters (i.e., difference between maximum and minimum RTTs) in OS-based virtualization are much less than those in hypervisor-based virtualization for all message sizes. The jitters for 4-byte messages, for example, were 6 μs with OS-based virtualization and 72 μs with hypervisor-based virtualization.

### 4.2. Analysis of Worst-Case End-to-End Delay

In Section 3, we suggested a hierarchical real-time scheduler and a simulator, which can improve the worst-case end-to-end delay by adjusting the phase offsets of virtual controllers and tasks. To measure the end-to-end delay, we ran virtual controllers with our driver-level CAN virtualization on a master node equipped with an Intel i5 processor and a two-port PEAK-System CAN interface. Two worker nodes equipped with an Intel i3 processor were connected to the different CAN ports of the master node. In the experiments, we ran two virtual controllers, $VC_{0}$ and $VC_{1}$ on the master node, each of which communicates to a worker node through a different CAN bus. Each worker node runs only a single virtual controller ($VC_{0}$ or $VC_{1}$). We consider the scenario in which we run the virtual controllers on general-purpose computing nodes provided by a cloud and have a capable of adjusting phase offsets on both master and worker nodes. As defined in Section 3.1, the end-to-end delay ($D_{e2e}$) is the time from the beginning of the sensing and control task at the master to the completion of the actuation task at the worker. Our measurements were done in three steps. First, we randomly generated three test sets as shown in Table 1. In each test set, we assumed that $\tau_{1}$ of $VC_{0}$ and $\tau_{0}$ of $VC_{1}$ performed either sensing and control or actuation (denoted as (sc) and (a) in Table 1, respectively). Then, we ran simulations for each test set to find a sub-optimal combination of phase offsets. The simulation parameters are shown in Table 2. Finally, we applied the sub-optimal combinations suggested by the simulator to our experimental system and measured the actual end-to-end delays of 1000 messages.

**Figure 11:**
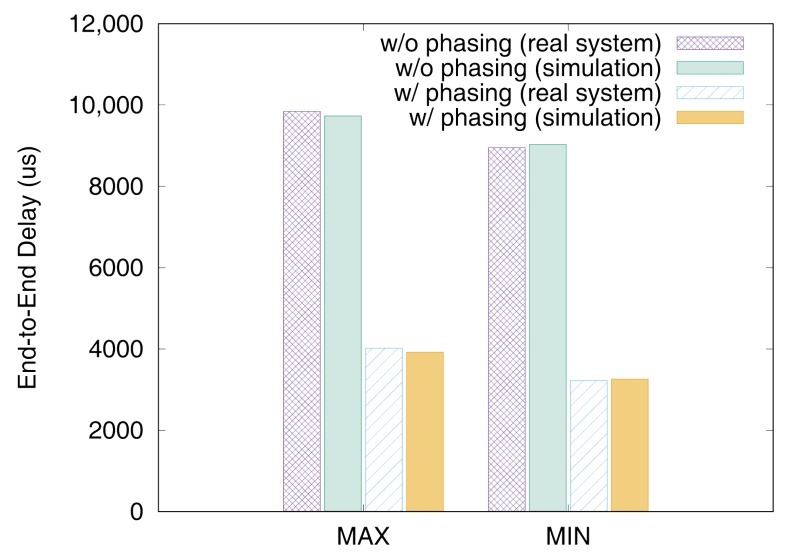
Max and min $D_{e2e}$ of $VC_{0}$ in test set 0.

**Figure 12:**
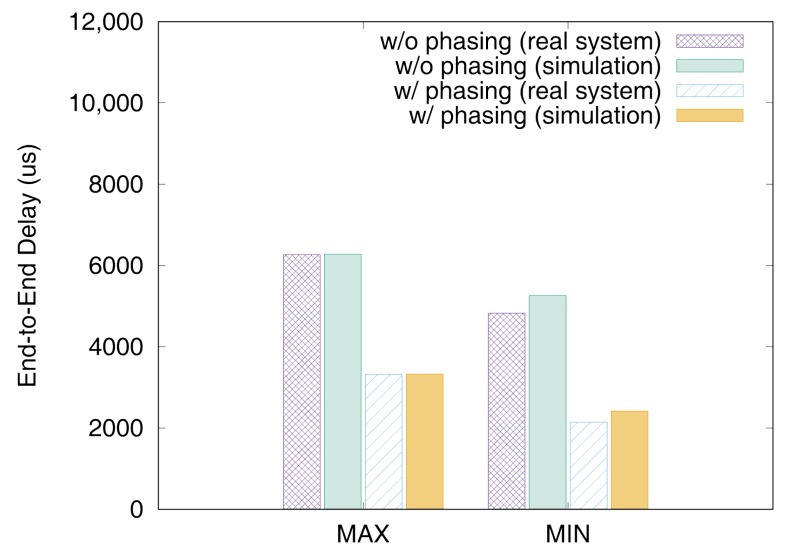
Max and min $D_{e2e}$ of $VC_{1}$ in test set 0.

**Figure 13:**
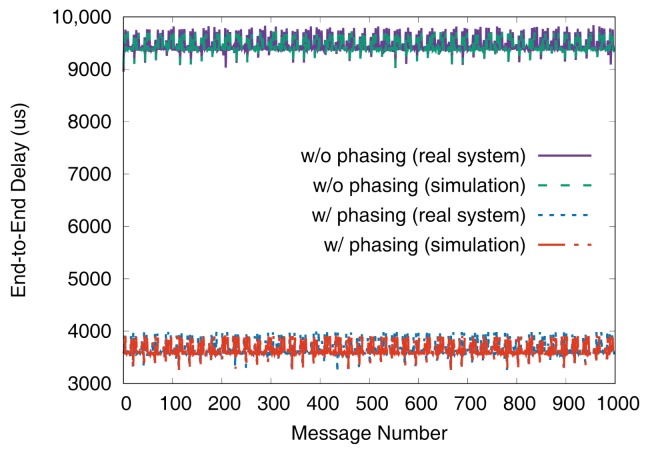
$D_{e2e}$ distribution of $VC_{0}$ in test set 0.

**Figure 14:**
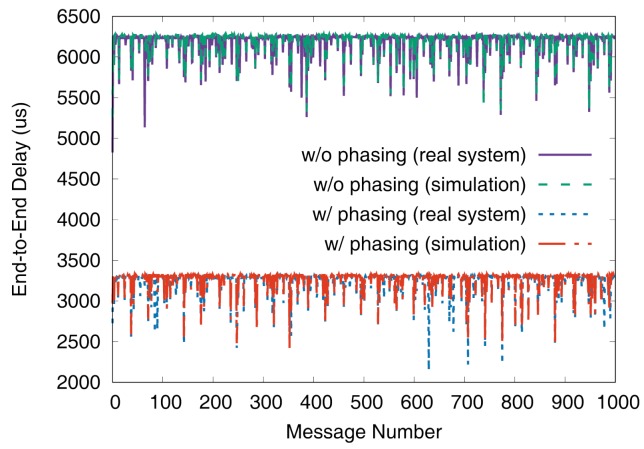
$D_{e2e}$ distribution of $VC_{1}$ in test set 0.

Figure 11 and Figure 12 show the end-to-end delays of $VC_{0}$ and $VC_{1}$ of test set 0, respectively. These graphs show the worst-case (denoted as MAX) and the best-case (denoted as MIN) delays and compare the values actually measured on a real system with those simulated to show that the errors by simulation are marginal (less than 2.5% for the worst-case end-to-end delay). As we can observe, phasing reduces the worst-case end-to-end delay by 59.2% for $VC_{0}$ and 47.0% for $VC_{1}$, respectively. Figure 13 and Figure 14 show the end-to-end delay of every iteration. Although the error is often seen for some specific iteration because the simulation environment (e.g., execution time of tasks) and the actual system environment may be different at those points, it can be seen that the overall maximum and the minimum values of the simulation results and the actual measurement results are similar.

**Figure 15:**
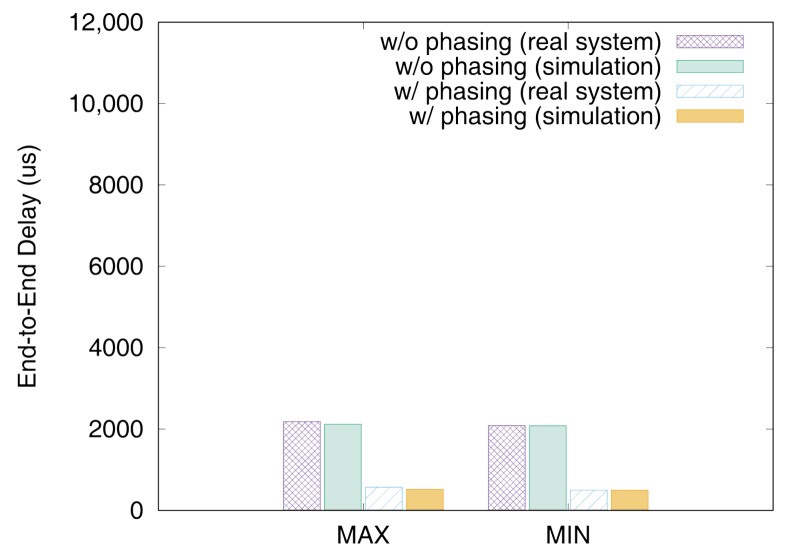
Max and min $D_{e2e}$ of $VC_{0}$ in test set 1.

**Figure 16:**
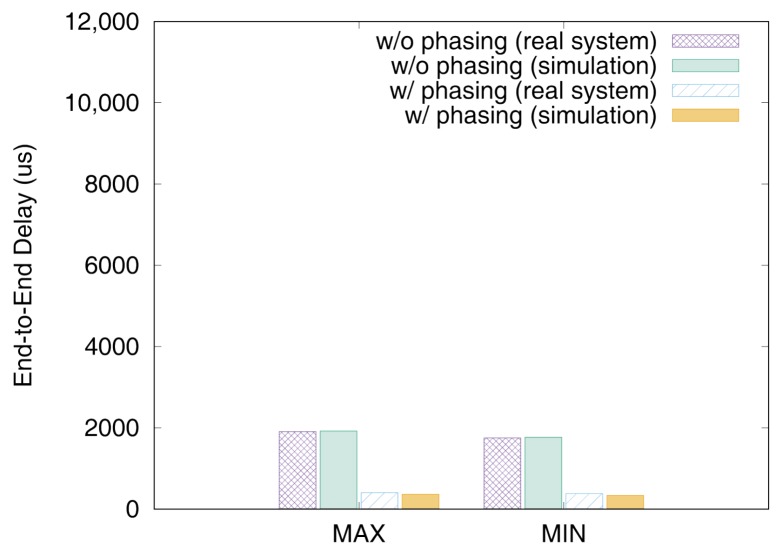
Max and min $D_{e2e}$ of $VC_{1}$ in test set 1.

**Figure 17:**
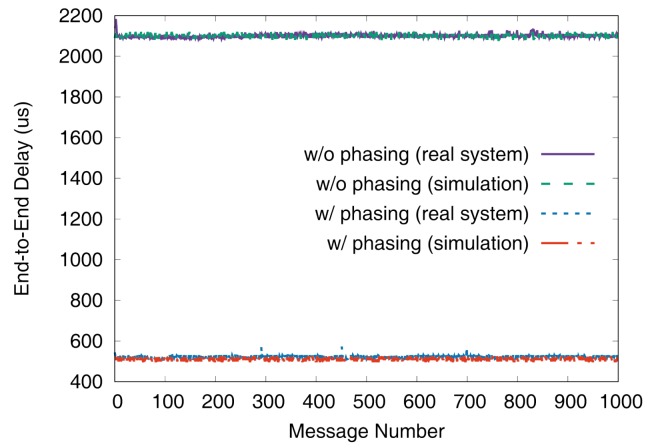
$D_{e2e}$ distribution of $VC_{0}$ in test set 1.

**Figure 18:**
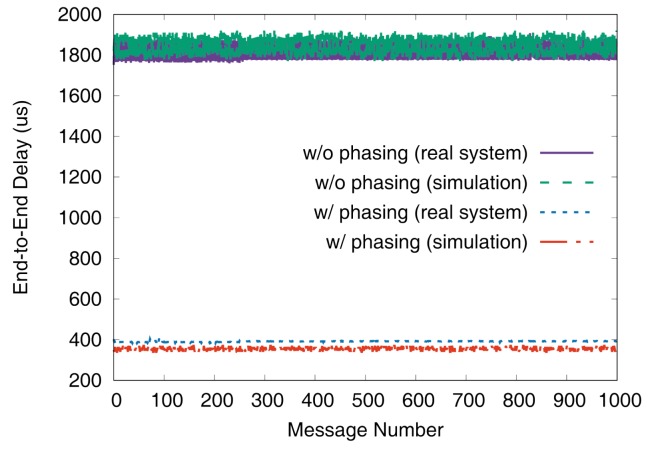
$D_{e2e}$ distribution of $VC_{1}$ in test set 1.

**Figure 19:**
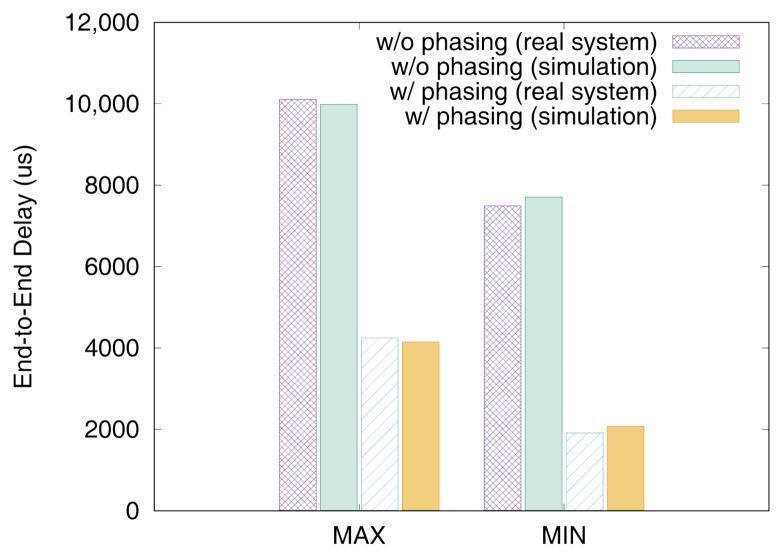
Max and min $D_{e2e}$ of $VC_{0}$ in test set 2.

**Figure 20:**
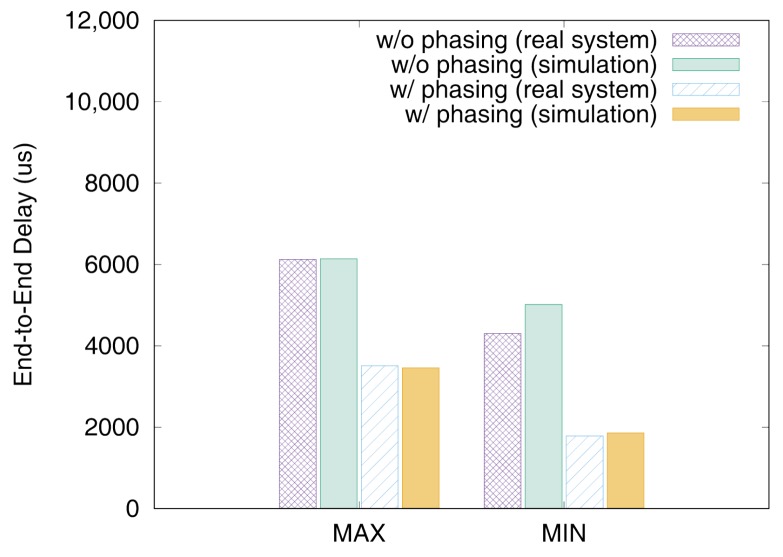
Max and min $D_{e2e}$ of $VC_{1}$ in test set 2.

**Figure 21:**
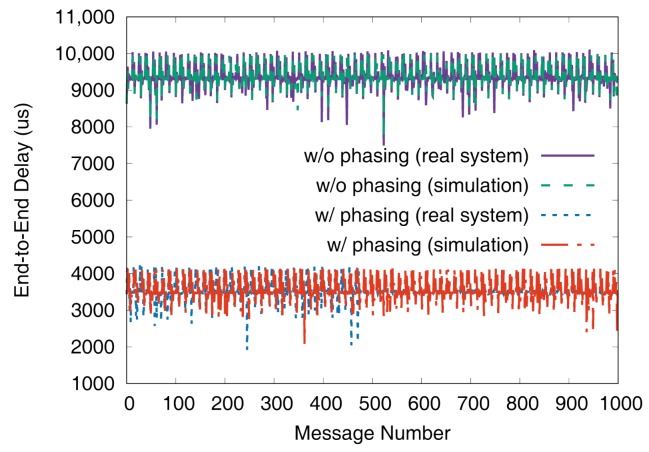
$D_{e2e}$ distribution of $VC_{0}$ in test set 2.

**Figure 22:**
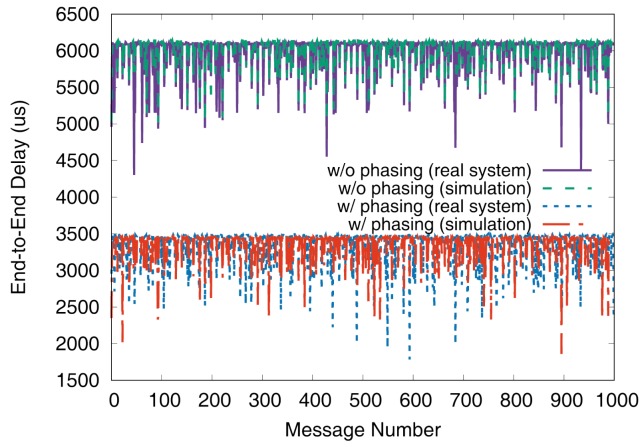
$D_{e2e}$ distribution of $VC_{1}$ in test set 2.

Figure 15, Figure 16, Figure 17, Figure 18, Figure 19, Figure 20, Figure 21 and Figure 22 show the measurement results for the test sets 1 and 2. We again see that the simulator can predict the worst-case end-to-end delays accurately and provide a sub-optimal phase combination successfully. In these experiments, the phasing scheme reduced the worst-case end-to-end delay on a real system up to 78.7% with test set 1 and 58.0% with test set 2, respectively.

## 5. Conclusions

The virtualization technologies can provide efficient hosting and consolidation of computing resources. Thus, several researchers tried to utilize the virtualization technologies in industrial control systems and showed benefits with respect to manageability and scalability. However, the support for traditional fieldbuses, such as CAN, in virtualized environments has not been studied thoroughly. In this paper, to tackle both functionality and QoS issues of CAN in virtualized environments, we suggested the lightweight CAN virtualization technology for containerized controllers. In the functionality aspect, our driver-level virtualization technology provided the abstractions for virtual CAN interfaces and virtual CAN buses, while preserving the transparency to system software and applications. In the QoS perspective, we provided a hierarchical real-time scheduler and phasing of virtual controllers and tasks. To provide a sub-optimal phase combination, we implemented a simulator. The experiment results showed that our CAN virtualization that targeted the OS-based virtualization had significantly lower overheads and less jitters compared with a hypervisor-based virtualized environment. In addition, we showed that the worst-case end-to-end delay could be reduced up to 78.7% by adjusting the phase offsets of virtual controllers and their tasks. As future work, we plan to apply our virtualization technology to a large-scale system that consists of more virtual controllers. To do this, we have to optimize the simulator, which currently takes several hours to generate a sub-optimal phase combination for the test cases discussed in this paper.

## Figures and Tables

**Table 1:** Test sets (time unit: μs).

Test Sets	Sets of *VC*s	Virtual Controllers	Period (Π)	Duration (Θ)	Tasks	Period (*p*)	Execution Time (*e*)
0	$C_{0}^{master}$	$VC_{0}$	2760	1000	$\tau_{0}$ $\tau_{1}\left(sc\right)$ $\tau_{2}$	5530 8800 9650	330 580 390
		$VC_{1}$	2970	1050	$\tau_{0}\left(sc\right)$ $\tau_{1}$ $\tau_{2}$	5950 7140 9980	310 490 550
	$C_{0}^{worker0}$	$VC_{0}$	100,000	95,000	$\tau_{0}$ $\tau_{1}\left(a\right)$ $\tau_{2}$	5530 8800 9650	330 580 390
	$C_{0}^{worker1}$	$VC_{1}$	100,000	95,000	$\tau_{0}\left(a\right)$ $\tau_{1}$ $\tau_{2}$	5950 7140 9980	310 490 550
1	$C_{1}^{master}$	$VC_{0}$	4000	1500	$\tau_{0}$ $\tau_{1}\left(sc\right)$ $\tau_{2}$	2000 2000 4000	60 100 110
		$VC_{1}$	4000	1500	$\tau_{0}\left(sc\right)$ $\tau_{1}$ $\tau_{2}$	2000 2000 4000	50 140 120
	$C_{1}^{worker0}$	$VC_{0}$	100,000	95,000	$\tau_{0}$ $\tau_{1}\left(a\right)$ $\tau_{2}$	2000 2000 4000	50 100 100
	$C_{1}^{worker1}$	$VC_{1}$	100,000	950,000	$\tau_{0}\left(a\right)$ $\tau_{1}$ $\tau_{2}$	2000 2000 4000	50 100 100
2	$C_{2}^{master}$	$VC_{0}$	2610	1100	$\tau_{0}$ $\tau_{1}\left(sc\right)$ $\tau_{2}$	5230 8840 9610	660 470 510
		$VC_{1}$	2840	1000	$\tau_{0}\left(sc\right)$ $\tau_{1}$ $\tau_{2}$	5680 10,580 11,090	440 650 420
	$C_{2}^{worker0}$	$VC_{0}$	100,000	95,000	$\tau_{0}$ $\tau_{1}\left(a\right)$ $\tau_{2}$	5230 8840 9610	660 470 510
	$C_{2}^{worker1}$	$VC_{1}$	100,000	95,000	$\tau_{0}\left(a\right)$ $\tau_{1}$ $\tau_{2}$	5680 10,580 11,090	440 650 420

**Table 2:** Simulation parameters.

Parameters	Value
Phasing resolution	50 μs
Simulation iterations	1000
Simulation resolution	10 μs
Interrupt handling overhead	20 μs
Tx and Rx queue size	10
Fieldbus bandwidth	1 Mbps
Fieldbus forwarding delay	1 μs
Message size	8 bytes
Direct Memory Access (DMA) overhead	10 μs

## Abbreviations

The following abbreviations are used in this manuscript:

CAN	Controller Area Network
CFD	Cumulative Distribution Function
cgroup	Control Group
DMA	Direct Memory Access
HMI	Human–Machine Interface
I/O	Input/Output
OS	Operating System
PLC	Programmable Logic Controller
QoS	Quality-of-Service
RM	Rate Monotonic
RTT	Round-Trip-Time
SCADA	Supervisory Control and Data Acquisition
SDN	Software-Defined Networking
TCP/IP	Transmission Control Protocol/Internet Protocol
VC	Virtual Controller
VMM	Virtual Machine Monitor
